# Divergent evolution of vitamin B9 binding underlies Juno-mediated adhesion of mammalian gametes

**DOI:** 10.1016/j.cub.2015.12.034

**Published:** 2016-02-08

**Authors:** Ling Han, Kaoru Nishimura, Hamed Sadat Al Hosseini, Enrica Bianchi, Gavin J. Wright, Luca Jovine

**Affiliations:** 1 Department of Biosciences and Nutrition & Center for Innovative Medicine, Karolinska Institutet, Huddinge, SE-141 83, Sweden; 2 Cell Surface Signalling Laboratory, Wellcome Trust Sanger Institute, Hinxton, Cambridge CB10 1SA, UK

## Abstract

The interaction between egg and sperm is the first necessary step of fertilization in all sexually reproducing organisms. A decade-long search for a protein pair mediating this event in mammals culminated in the identification of the glycosylphosphatidylinositol (GPI)-anchored glycoprotein Juno as the egg plasma membrane receptor of sperm Izumo1 1, 2. The Juno–Izumo1 interaction was shown to be essential for fertilization since mice lacking either gene exhibit sex-specific sterility, making these proteins promising non-hormonal contraceptive targets 1, 3. No structural information is available on how gamete membranes interact at fertilization, and it is unclear how Juno — which was previously named folate receptor (FR) 4, based on sequence similarity considerations — triggers membrane adhesion by binding Izumo1. Here, we report the crystal structure of Juno and find that the overall fold is similar to that of FRα and FRβ but with significant flexibility within the area that corresponds to the rigid ligand-binding site of these *bona fide* folate receptors. This explains both the inability of Juno to bind vitamin B_9_/folic acid [1], and why mutations within the flexible region can either abolish or change the species specificity of this interaction. Furthermore, structural similarity between Juno and the cholesterol-binding Niemann-Pick disease type C1 protein (NPC1) suggests how the modified binding surface of Juno may recognize the helical structure of the amino-terminal domain of Izumo1. As Juno appears to be a mammalian innovation, our study indicates that a key evolutionary event in mammalian reproduction originated from the neofunctionalization of the vitamin B_9_-binding pocket of an ancestral folate receptor molecule.

## Teaser

Recent studies have shown that the GPI-anchored glycoprotein Juno is the egg plasma membrane receptor of sperm Izumo1. Here, Han *et al.* report the crystal structure of Juno and find that its overall fold is similar to that of folate receptors but with important differences in the ligand-binding site.

## Main Text

To gain insights into how Juno recognizes Izumo1, we expressed the complete ectodomain of mouse Juno (residues G20–A221) as a soluble protein in glycosylation-impaired mammalian cells (see Supplemental Experimental Procedures). Following enzymatic trimming of N-glycans, affinity-purified Juno behaved as a monomer by size-exclusion chromatography (Figure S1A,B) and yielded a ∼10 μm thick crystal that diffracted to 2.7 Å resolution (Figure S1C). The structure of Juno was solved by molecular replacement and refined to R = 23.3%, Rfree = 24.8% (Figure S1D–F and PDB ID 5EJN).

**Figure 1:**
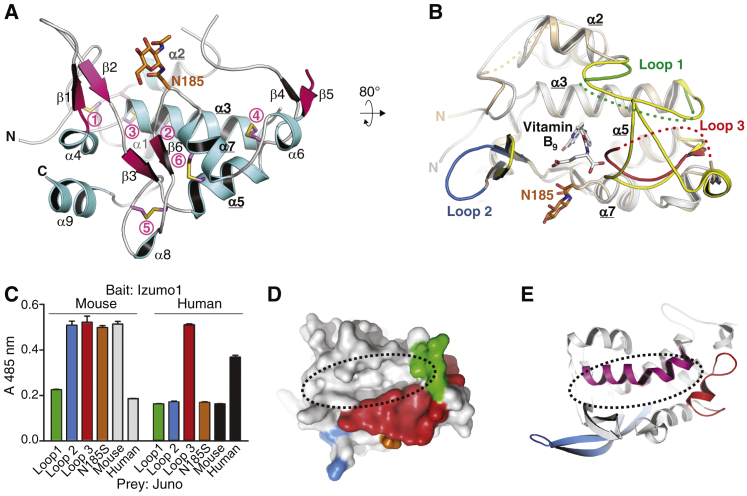
Experimental results and proposed mode of Juno–Izumo1 interaction. (A) Crystal structure of mouse Juno, depicted in cartoon representation and colored according to secondary structure. Conserved disulfides (circled pink numbers, see also Figure S2A) and the N-acetylglucosamine (GlcNAc) residue attached to N185 are shown as sticks and colored pink/yellow and brown, respectively. Amino/carboxyl termini and secondary structure elements are marked, with core α-helix labels highlighted in bold and underlined. (B) Superposition of mouse Juno (orange) and human FRα bound to vitamin B_9_ (grey; PDB ID 4LRH[4]) highlights structurally variant regions. Juno loop 1, located between α-helices 2 and 3, is green; loop 2, overlapping with the inhibitory loop of FRs [5], is blue; loop 3, between β-strands 4 and 5, is red. Juno loop 1 and 3 residues lacking electron density are indicated by dotted lines; regions of FRα corresponding to Juno loops 1–3 are yellow. Core helices are marked, and Juno N185 GlcNAc is shown as in (A). (C) AVEXIS identifies loops 1 and 3 of Juno as important for interaction with Izumo1. Mutant mouse Juno proteins in which loops 1–3 or N185 were replaced with the corresponding human residues (see Supplemental Experimental Procedures) were expressed as preys and tested for their ability to bind immobilized Izumo1 bait proteins. Bars represent means ± s.e.m.; n = 3. (D) Structural mapping of functionally important loops 1 and 3 reveals a groove on the surface of Juno (dotted black oval). Juno is shown in surface representation, approximately oriented as in (B), with loops 1–3 and N185 colored as in (B,C). (E) In structural homologue NPC1 (PDB ID 3GKI[9]), an additional α-helix (residues D76–S95; purple) occupies the region corresponding to the groove of Juno. A cartoon model of NPC1 is shown, oriented as in (D) upon superimposition on Juno (Figure S2B). NPC1 regions corresponding to Juno loops 2 and 3 are colored as in (B–D).

Juno consists of nine α-helices and six short β-strands (Figure 1A and Figure S2A), which adopt the same fold of FRα and FRβ 4, 5 and riboflavin-binding protein (RfBP) [6]. The bulk of the structure, including four core α-helices and seven disulfide bonds, can be superimposed onto the ligand-bound form of human FRα [4] and FRβ [5] with root mean square deviations (RMSD) of 1.4 Å and 1.6 Å over 166 and 167 residues, respectively (Figure 1B). Although glycosylation at N73 is important for secretion of Juno (Figure S1G, lane 1), there is no electron density for the loop carrying the corresponding GlcNAc, suggesting that this conserved carbohydrate affects protein solubility rather than folding. A crystal contact, however, fixes the position of the N-glycan at N185 (Figure S1F), which is neither conserved (Figure S2A) nor required for secretion (Figure S1G, lane 2).

Despite their overall similarity, there are striking local differences between the structure of Juno and that of FRs and RfBP. Whereas in the latter proteins three highly ordered loops generate a deep binding pocket for the respective ligands 4, 5, 6, the corresponding regions of Juno are either largely disordered (loops 1 and 3) or adopt a very different conformation (loop 2) (Figure 1B and Figure S2A). Notably, the structured nature of these loops in FRs does not depend on vitamin B_9_ binding because they are also visible in the unbound forms of FRα and FRβ [5]. Taken together with sequence alignments (Figure S2A), these observations suggest that — in addition to key amino acid substitutions such as D103A 1, 4 — structural disorder or displacement of conserved pocket residues important for ligand recognition in FRα [4] contribute to the inability of Juno to bind vitamin B_9_. These amino acids include FRα F84 and G159–W162, whose counterparts in loops 1 and 3 of Juno are completely disordered (Figure S2A), as well as FRα R128, which in Juno is shifted by ∼13 Å because of the different conformation of the loop 2 region (Figure 1B).

Although the Juno–Izumo1 interaction is conserved in mammals [1], it demonstrates some specificity across species. For example, mouse Juno does not bind human Izumo1, as assessed by avidity-based extracellular interaction screen (AVEXIS), a sensitive extracellular protein interaction assay (Figure 1C) [7]. Interestingly, the sequence identity between mouse and human Juno (70% overall) drops in loops 1 and 2, as well as in the disordered carboxy-terminal half of loop 3 (53%, 12% and 47%, respectively; Figure S2A). To test whether these regions of Juno are involved in its species-specific interaction with Izumo1, we designed a series of mouse Juno protein variants where loops 1–3 were individually replaced with the corresponding human sequences (Figure S2A). Additionally, an N185S mutant was also produced to assess whether the non-conserved N-glycan of Juno, which is located close to loop 2 (Figure 1A,B), is important for mouse Izumo1 recognition. Using AVEXIS we showed that, whereas loop 2 and N185S mutant mouse Juno proteins still bound mouse Izumo1, binding was lost upon humanization of loop 1. Moreover, humanization of loop 3 altered the species specificity of mouse Juno, so that it additionally bound human Izumo1 (Figure 1C). Together, these results suggest that the area delimited by loops 1 and 3 mediates binding of Juno to Izumo1, and that the flexible parts of these loops may become ordered upon protein–protein interaction.

Although the structure of Izumo1 is unknown, its amino-terminal Juno-binding region has been shown to be helical [8]. Notably, loops 1 and 3 of Juno surround a groove in the protein surface (Figure 1D), and the corresponding region of NPC1 [9] — a distant structural homologue of Juno (Figure S2B) — accommodates an additional α-helix (Figure 1E). Based on these considerations, it can be hypothesized that docking of an α-helix of Izumo1 onto the groove of Juno may underlie adhesion of the gamete plasma membranes.

Whereas FR homologues are found in all vertebrate classes, Juno appears restricted to mammals. Together with this observation, our studies suggest that the molecular basis of mammalian gamete recognition evolved from an ancestral FR that lost the ability to bind vitamin B_9_, but gained the ability to recognize Izumo1.

## Author Contributions

Conceptualization, K.N., G.J.W. and L.J.; methodology, L.H., K.N., L.J.; validation, E.B.; formal analysis, K.N., L.J.; investigation, L.H., K.N., H.S.A.H, E.B.; data curation, K.N., L.J.; writing – original draft, L.H., K.N., L.J.; writing – review and editing, L.H., K.N., H.S.A.H, E.B., G.J.W., L.J.; visualization, K.N., E.B., L.J.; supervision, G.J.W., L.J.; project administration, L.H., K.N., L.J.; funding acquisition, G.J.W., L.J.

## Supplemental Information

Document S1. Two Figures and Experimental Procedures
