# Supplementary material for: Divergent evolution of vitamin B9 binding underlies Juno-mediated adhesion of mammalian gametes
Source: Curr Biol. 2016 Feb 8;26(3):R100–1. doi: 10.1016/j.cub.2015.12.034 (PMC4751342; doi:10.1016/j.cub.2015.12.034)
Supplement: Document S1. Two Figures and Experimental Procedures [file mmc1.pdf]

Supplemental Information

Divergent evolution of vitamin B_9 binding underlies Juno-mediated adhesion of mammalian gametes

Ling Han, Kaoru Nishimura, Hamed Sadat Al Hosseini, Enrica Bianchi, Gavin J. Wright, and Luca Jovine

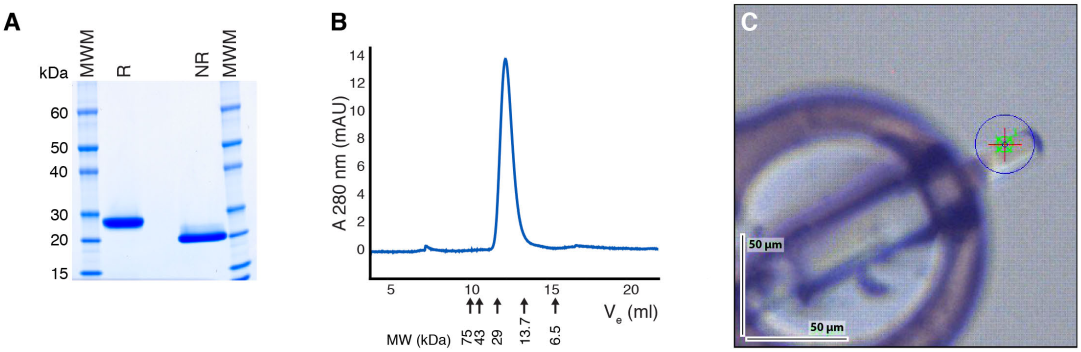

D

Crystal (PDB ID)	Juno (5EJN)
**Experiment**	
Beamline	ESRF ID29
Wavelength (Å)	0.984
**Data Collection**	
Space group	*P* 1 2_1 1 (4)
Cell dimensions	
a, b, c (Å)	38.1, 52.2, 87.5
α, β, γ (°)	90, 90.0, 90
Molecules / A.U.	2
Solvent content (%)	28.4
Mosaicity (°)	0.257
Wilson B factor (Å^2)	72.7
Resolution (Å)	44.83 - 2.70 (2.80 - 2.70)
Total reflections	44480 (3965)
Unique reflections	9510 (933)
Completeness (%)	99.3 (98.9)
Redundancy	4.7 (4.2)
Mean I/σ(I)	13.7 (1.9)
CC(1/2)	0.99 (0.57)
R_{meas} (%)	8.1 (88.2)
R_{pim} (%)	3.7 (41.4)

**Refinement**	
Resolution (Å)	44.83 - 2.70
Reflections	9510
Free reflections	482
Twin operator / fraction	h, –k, –l (0.49)
R_{work} / R_{free} (%)	23.30 / 24.82
CC_{work} / CC_{free}	0.89 / 0.91 (0.51 / 0.42)
ML coordinate error (Å)	None
ML phase error (°)	39.25
R.m.s. deviations	
Bond lengths (Å)	0.003
Bond angles (°)	0.650
Ramachandran plot	
Favored (%)	98.12
Allowed (%)	1.88
Outlier (%)	0.00
Number of atoms	
Total	2796
Protein	2782
Carbohydrate	14
Protein residues	338
Average B factor (Å^2)	
Total	103.25
Protein	103.31
Carbohydrate	91.38

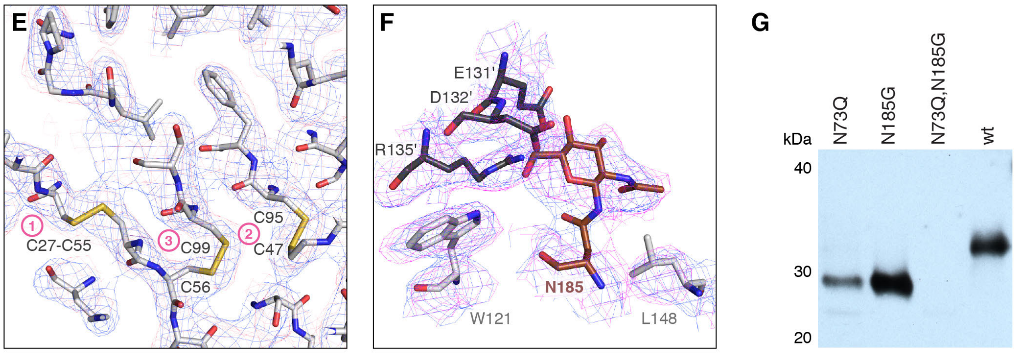

Figure S1

Figure S1. Characterization and structure determination of recombinant Juno.

(A) SDS-PAGE analysis of purified mouse Juno. The relative shift in migration between samples (4 µg) run under reducing (R) and non-reducing (NR) conditions is due to the presence of 8 conserved intramolecular disulfides bonds (Figure 1A and Figure S2A). MWM, molecular weight marker. (B) Analytical size-exclusion chromatography (SEC) analysis of purified Juno, whose elution volume (V_e) is consistent with a monomeric state. Arrows below the chromatogram mark the V_e of protein standards of the indicated molecular weights (MW). (C) Juno crystal used for data collection. The size of the X-ray beam is indicated by the blue circle. (D) X-ray data collection and refinement statistics. (E) $2F_{obs}$-DF_{calc} likelihood-weighted map (blue mesh) and feature-enhanced map (pink mesh) of the region around disulfides 1–3. Both electron density maps are uncarved and contoured at 1 σ. (F) The GlcNAc residue attached to N185 of Juno molecule B (brown) interacts with W121 and L148 of the same molecule (light grey) and is involved in a crystal contact with a symmetry-related copy of chain B (dark grey). Maps are as in (E), but were carved around displayed residues for clarity. (G) Reducing anti-5His immunoblot of 20 µl conditioned medium of HEK293-T cells expressing fully glycosylated wt mouse Juno and different glycosylation mutants thereof.

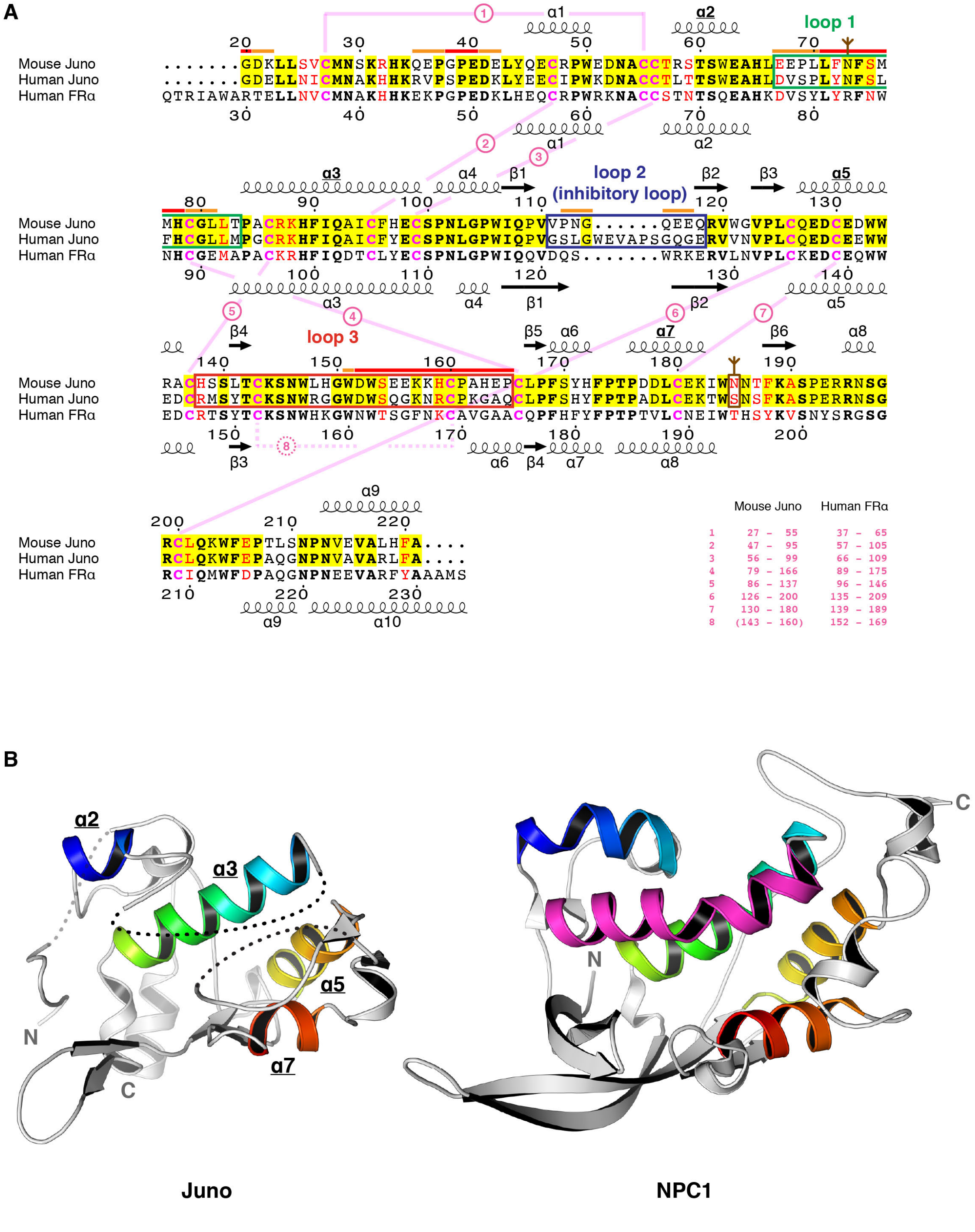
A
loop 1
loop 2
(inhibitory loop)
loop 3
Mouse Juno .......GDKLLSVCMNSKRHKQEPGPEDELYQECRPWEDNACCTRSTSWEAHLEEPLLFNFSM
Human Juno .......GDELLNICMNAKHHKRVPSPEDKLYEECIPWKDNACCTLTTSWEAHLDVSPLYNFSL
Human FRα QTRIAWARTELLNVCMNAKHHKEKPGPEDKLHEQCRPWRKNACCSTNTSQEAHKDVSYLYRFNW
Mouse Juno MHCGLLTPACRKHFIQAICFHECSPNLGPWIQPVVPNG......QEEQRVWGVPLCQEDCEDWW
Human Juno FHCGLLMPGCRKHFIQAICFYECSPNLGPWIQPVGSLGWEVAPSGQGERVVNVPLCQEDCEEWW
Human FRα NHCGEMAPACKRHFIQDTCLYECSPNLGPWIQQVDQS.......WRKERVLNVPLCKEDCEQWW
Mouse Juno RACHSSLTCKSNWLHGWDWSEEKKHCPAHEPCLPFSYHFPTPDDLCEKIWNNTFKASPERRNSG
Human Juno EDCRMSYTCKSNWRGGWDWSQGKNRCPKGAQCLPFSHYFPTPADLCEKTWSNSFKASPERRNSG
Human FRα EDCRTSYTCKSNWHKGWNWTSGFNKCAVGAACQPFHFYFPTPTVLCNEIWTHSYKVSNYSRGSG
Mouse Juno RCLQKWFEPTLSNPNVEVALHFA....
Human Juno RCLQKWFEPAQGNPNVAVARLFA....
Human FRα RCIQMWFDPAQGNPNEEVARFYAAAMS
Mouse Juno Human FRα
1 27 - 55 37 - 65
2 47 - 95 57 - 105
3 56 - 99 66 - 109
4 79 - 166 89 - 175
5 86 - 137 96 - 146
6 126 - 200 135 - 209
7 130 - 180 139 - 189
8 (143 - 160) 152 - 169
B
α2
α3
α5
α7
N
C
Juno
NPC1

Figure S2

Figure S2. Features of Juno and sequence/structure alignment to FRα and NPC1.

(A) Structure-based sequence alignment of Juno homologues and human FRα. Residues conserved between mouse and human Juno are highlighted in yellow; amino acids identical or similar in all sequences are bold or red, respectively. Secondary structure elements of mouse Juno and human FRα are indicated above and below the sequences, respectively, with core helices labelled as in Figure 1A. Conserved disulfide bonds 1–8 are indicated by pink lines, with corresponding cysteine pair numbers reported in the table on the bottom right. Juno disulfide bond 8 is not defined in the electron density, and has thus been assigned by exclusion and based on similarity with FRα. Juno N-glycosylation sites are indicated by inverted brown tripods, and regions corresponding to loop 1–3 as well as N185 are marked with boxes colored as in Figure 1B–D. Amino acids disordered in either Juno molecule A or B are indicated by an orange bar above their sequence, residues disordered in both Juno molecules are marked by a red bar. (B) Juno and NPC1 are structural homologues. Despite having only 12% sequence identity, the two molecules are clearly identified as being structurally similar by Dali (Z-score 7.5) and can be superimposed with an RMSD of 3.1 Å over 109 residues. The four core α-helices of Juno and the corresponding helices of NPC1 (PDB ID 3GKI [S1]) (α1: P52-L62; α3: C97-S114; α5: G149-R161; α7: Q185-F194) are shown in rainbow color; the extra helix of NPC1 (α2: D76-S95) is shown in purple, as in Figure 1E. For clarity, molecules have been translated relative to each other after superposition.

SUPPLEMENTAL EXPERIMENTAL PROCEDURES

DNA Constructs

Carboxy-terminally 8His-tagged Juno expression constructs for crystallographic studies were obtained by cloning synthetic genes (DNA2.0; GeneArt/Life Technologies) into pHLsec3H, a mammalian expression vector derived from pHLsec [S2]. Constructs expressing N-glycosylation site mutants of Juno were generated by overlap extension PCR with reverse-phase cartridge-purified oligonucleotides (SIGMA-ALDRICH) and PfuTurbo DNA polymerase (Agilent Technologies), followed by cloning into pHLsec3H and verification by DNA sequencing (Eurofins Genomics). Juno and Izumo1 ectodomain constructs for AVEXIS experiments were generated by cloning synthetic genes (GeneArt/Life Technologies) into expression vectors that encoded either biotinylated 'baits' or pentameric β-lactamase-tagged 'preys' [S3]. In mouse Juno loop mutants 1, 2 and 3, sequences encoding residues E67–T83, V111–Q118 and H138–P165 were replaced with the corresponding sequences of human Juno (encoding D67–M83, G111–E124 and R144–Q171, respectively). In mutant 4, the N-glycosylation site at position 185 of mouse Juno was inactivated by mutation to the corresponding residue of the human homolog (S191). DNA for transfection was prepared using HiSpeed Plasmid Midi kits or EndoFree Plasmid Giga kits (QIAGEN).

Protein Expression

For structural studies N-acetylglucosaminyltransferase I-deficient ($GnTI^{-}$) cell line Human Embryonic Kidney (HEK) 293-S (ATCC CRL-3022) was used, which secretes proteins carrying Endoglycosidase H (Endo H)-sensitive $Man_5GlcNAc_2$ N-glycans [S4,S5]. Cells were cultivated in Dulbecco's Modified Eagle Medium (DMEM; Life Technologies) supplemented with 10% fetal bovine serum (Saveen Werner) at 37ºC, 5% CO_2. Transient transfections were performed in DMEM supplemented with 4 mM L-glutamine using 25 kDa branched polyethyleneimine (SIGMA-ALDRICH), essentially as described [S2]. Small- and large-scale transfections were carried out in 6-well plates (10 cm^2/well; Corning) and cell factories (2528 cm^2; Nunc), respectively. Proteins for N-glycosylation site analysis (Figure S1G) and AVEXIS assays (Figure 1C) were expressed using cell lines HEK293-T (a kind gift from R. Aricescu, University of Oxford) and HEK293-6E [S6], respectively.

Protein Purification

Secreted proteins were captured from conditioned medium by batch immobilized metal-affinity chromatography with Ni-NTA Superflow (QIAGEN). After Endo H treatment, proteins were subjected to SEC using a HiLoad 26/60 Superdex 75 column (GE Healthcare) equilibrated against 10 mM HEPES pH 7.8, 150 mM NaCl and finally concentrated to 6 mg/ml. Analysis of the oligomeric state of purified Juno (Figure S1A) was performed using a calibrated Superdex 75 10/300 GL analytical SEC column (GE Healthcare) (Figure S1B).

Western Blot Analysis

Immunoblot experiments were performed using Penta-His mouse monoclonal antibody (QIAGEN; 1:1000) and peroxidase-conjugate AffiniPure goat anti-mouse IgG (Jackson ImmunoResearch Lab; 1:10,000). Chemiluminescence detection was performed with Western Lightning ECL Plus (Perkin Elmer).

Protein Crystallization

Clusters of thin rod-shaped crystals (~200 μm long) were grown by vapor diffusion against mother liquor containing 200 mM $CaCl_2$, 100 mM Tris-HCl pH 8.5, 25% (w/v) PEG 4000 at 20ºC. Individual crystals were obtained by breaking clusters with a microtool, cryoprotected using mother liquor supplemented with 5% PEG 200 and 5% PEG 4000, mounted in MicroLoops (MiTeGen) and flash cooled in liquid nitrogen prior to data collection at 100 K.

X-ray Diffraction Data Collection, Processing and Structure Determination

Of more than one hundred crystals screened at synchrotron, only one specimen (Figure S1C) yielded usable X-ray diffraction data (Figure S1D). This was collected using a PILATUS 6M-F detector (DECTRIS) at European Synchrotron Radiation Facility (ESRF) beamline ID29 [S7]. Integration and scaling were performed with XDS [S8], using CC(1/2) = 0.5 as high resolution cutoff criterion [S9]. A search ensemble for molecular replacement (MR) was generated using MrBUMP [S10], based on chains E–H of human FRα (PDB ID 4LRH; 52.7% sequence identity to Juno) [S11]. A single MR solution with

one molecule per asymmetric unit was found by PHASER [S12] in space group $P2_12_12$ (translation function Z-score 17.0), which was autotraced with PHENIX AutoBuild [S13] and manually rebuilt in Coot [S14]. Refinement of the resulting model with phenix.refine [S15] could however not proceed beyond R_{free} ~32.5%. This suggested that the initial space group assignment was incorrect, and that the crystal belonged to space group $P2_1$ with β ~90° and a high merohedral twin fraction that mimicked primitive orthorhombic symmetry. Refinement of the model in $P2_1$, with twinning operator h, –k, –l and two molecules of Juno per asymmetric unit, yielded a final R_{free} value of 24.8% (Figure S1D). Chains A and B of the final model contain 166 and 172 amino acids, respectively. Juno residues G20, Q35–E40, E67–H78, P112–E117, D152–P165 of molecule A and G20–K22, G38–E42, L71–L81, W151–P165 of molecule B are not defined in the electron density map (Figure S2A); molecule A L43 and molecule B R194 also have very poor density, but were modelled because their position is constrained by the neighboring, well-defined residues. Feature-enhanced maps were used for rebuilding in addition to conventional σA-weighted maps [S16]; protein geometry was validated with MolProbity [S17] and carbohydrate structure validation was carried out using Privateer [S18]. Structure comparisons were performed using PDBeFold [S19], pairwise DaliLite [S20] and the Dali server [S21]. Structural figures were prepared with PyMOL (Schrödinger); the alignment in Figure S2A was generated using ESPript [S22]. Structure factor intensities and atomic coordinates have been deposited in the Protein Data Bank with accession code 5EJN.

Juno-Izumo1 Interaction Assay by AVEXIS

AVEXIS assays were performed essentially as described [S23]. Briefly, biotinylated baits were extensively dialyzed against PBS and both baits and preys were normalized as previously described [S24]. Biotinylated baits were immobilized in streptavidin-coated 96-well microtitre plates (NUNC) and washed, after which normalized preys were added and incubated for one hour. After washing three times with PBS/0.1% Tween-20, 1X PBS, colorimetric β-lactamase substrate nitrocefin was added at 125 μg/ml. Absorbance values were measured at 485 nm on a Pherastar plus (BMG Laboratories). A representative of four experiments is shown in Figure 1C, and the same conclusions were reached from an independent experiment where bait-prey orientations were reversed.

SUPPLEMENTAL REFERENCES

S1. Kwon H.J., Abi-Mosleh L., Wang M.L., Deisenhofer J., Goldstein J.L., Brown M.S., and Infante R.E. (2009). Structure of N-terminal domain of NPC1 reveals distinct subdomains for binding and transfer of cholesterol. Cell *137*, 1213-1224.

S2. Aricescu A.R., Lu W., and Jones E.Y. (2006). A time- and cost-efficient system for high-level protein production in mammalian cells. Acta Crystallogr. D Biol. Crystallogr. *62*, 1243-1250.

S3. Sun Y., Gallagher-Jones M., Barker C., and Wright G.J. (2012). A benchmarked protein microarray-based platform for the identification of novel low-affinity extracellular protein interactions. Anal. Biochem. *424*, 45-53.

S4. Reeves P.J., Callewaert N., Contreras R., and Khorana H.G. (2002). Structure and function in rhodopsin: high-level expression of rhodopsin with restricted and homogeneous N-glycosylation by a tetracycline-inducible N-acetylglucosaminyltransferase I-negative HEK293S stable mammalian cell line. Proc. Natl. Acad. Sci. USA *99*, 13419-13424.

S5. Chang V.T., Crispin M., Aricescu A.R., Harvey D.J., Nettleship J.E., Fennelly J.A., Yu C., Boles K.S., Evans E.J., Stuart D.I., *et al.* (2007). Glycoprotein structural genomics: solving the glycosylation problem. Structure *15*, 267-273.

S6. Loignon M., Perret S., Kelly J., Boulais D., Cass B., Bisson L., Afkhamizarreh F., and Durocher Y. (2008). Stable high volumetric production of glycosylated human recombinant IFNalpha2b in HEK293 cells. BMC Biotechnol. *8*, 65.

S7. de Sanctis D., Beteva A., Caserotto H., Dobias F., Gabadinho J., Giraud T., Gobbo A., Guijarro M., Lentini M., Lavault B., *et al.* (2012). ID29: a high-intensity highly automated ESRF beamline for macromolecular crystallography experiments exploiting anomalous scattering. J. Synchrotron Radiat. *19*, 455-461.

S8. Kabsch W. (2010). XDS. Acta Crystallogr. D Biol. Crystallogr. *66*, 125-132.

S9. Evans P.R., and Murshudov G.N. (2013). How good are my data and what is the resolution? Acta Crystallogr. D Biol. Crystallogr. *69*, 1204-1214.

S10. Keegan R.M., and Winn M.D. (2008). MrBUMP: an automated pipeline for molecular replacement. Acta Crystallogr. D Biol. Crystallogr. *64*, 119-124.

S11. Chen C., Ke J., Zhou X.E., Yi W., Brunzelle J.S., Li J., Yong E.L., Xu H.E., and Melcher K. (2013). Structural basis for molecular recognition of folic acid by folate receptors. Nature *500*, 486-489.

S12. McCoy A.J., Grosse-Kunstleve R.W., Adams P.D., Winn M.D., Storoni L.C., and Read R.J. (2007). Phaser crystallographic software. J. Appl. Crystallogr. *40*, 658-674.

S13. Terwilliger T.C., Grosse-Kunstleve R.W., Afonine P.V., Moriarty N.W., Zwart P.H., Hung L.W., Read R.J., and Adams P.D. (2008). Iterative model building, structure refinement and density modification with the PHENIX AutoBuild wizard. Acta Crystallogr. D Biol. Crystallogr. *64*, 61-69.

S14. Emsley P., Lohkamp B., Scott W.G., and Cowtan C. (2010). Features and development of Coot. Acta Crystallogr. D Biol. Crystallogr. *66*, 486-501.

S15. Afonine P.V., Grosse-Kunstleve R.W., Echols N., Headd J.J., Moriarty N.W., Mustyakimov M., Terwilliger T.C., Urzhumtsev A., Zwart P.H., and Adams P.D. (2012). Towards automated crystallographic structure refinement with phenix.refine. Acta Crystallogr. D Biol. Crystallogr. *68*, 352-367.

S16. Afonine P.V., Moriarty N.W., Mustyakimov M., Sobolev O.V., Terwilliger T.C., Turk D., Urzhumtsev A., and Adams P.D. (2015). FEM: feature-enhanced map. Acta Crystallogr. D Biol. Crystallogr. *71*, 646-666.

S17. Chen V.B., Arendall W.B., Headd J.J., Keedy D.A., Immormino R.M., Kapral G.J., Murray L.W., Richardson J.S., and Richardson D.C. (2010). MolProbity: all-atom structure validation for macromolecular crystallography. Acta Crystallogr. D Biol. Crystallogr. *66*, 12-21.

S18. Agirre J., Iglesias-Fernandez J., Rovira C., Davies G.J., Wilson K.S., and Cowtan K.D. (2015). Privateer: software for the conformational validation of carbohydrate structures. Nat. Struct. Mol. Biol. *22*, 833-834.

S19. Krissinel E., and Henrick K. (2005). Multiple Alignment of Protein Structures in Three Dimensions. In Lecture Notes in Computer Science, (Berlin, Heidelberg: Springer Berlin Heidelberg), pp. 67-78.

S20. Hasegawa H., and Holm L. (2009). Advances and pitfalls of protein structural alignment. Curr. Opin. Struct. Biol. *19*, 341-348.

S21. Holm L., and Rosenstrom P. (2010). Dali server: conservation mapping in 3D. Nucleic Acids Res. *38*, W545-W549.

S22. Gouet P., Robert X., and Courcelle E. (2003). ESPript/ENDscript: Extracting and rendering sequence and 3D information from atomic structures of proteins. Nucleic Acids Res. *31*, 3320-3323.

S23. Bushell K.M., Söllner C., Schuster-Boeckler B., Bateman A., and Wright G.J. (2008). Large-scale screening for novel low-affinity extracellular protein interactions. Genome Res. *18*, 622-630.

S24. Kerr J.S., and Wright G.J. (2012). Avidity-based extracellular interaction screening (AVEXIS) for the scalable detection of low-affinity extracellular receptor-ligand interactions. J. Vis. Exp. e3881.
